# The distribution and the clinical importance of MUC5B and TERT variants in Turkish patients with idiopathic pulmonary fibrosis

**DOI:** 10.1186/s12890-025-03903-7

**Published:** 2025-09-25

**Authors:** Ayşe Ödemiş, Aliye Candan Öğüş, Aslı Toylu, Hülya Dirol, Aykut Çilli, Ömer Özbudak, Özden Altıok Clark, Tülay Özdemir

**Affiliations:** 1 Faculty of Medicine, Department of Chest Diseases, Akdeniz University, Antalya, Türkiye Turkey; 2 Faculty of Medicine, Department of Medical Genetics, Akdeniz University, Antalya, Türkiye Turkey

**Keywords:** Mucine-5B (*MUC5B*), Telomerase reverse transcriptase (*TERT*), Idiopathic pulmonary fibrosis (IPF), Genetic, Variant, Fibrosis

## Abstract

**Background:**

The role of genetic variants in Mucin-5B (MUC5B) and telomerase reverse transcriptase (TERT) in idiopathic pulmonary fibrosis (IPF) pathogenesis, as well as their associations with clinical characteristics, remain uncertain and may exhibit ethnic variations.

**Methods:**

This single-center, cross-sectional study aimed to investigate the distribution of MUC5B rs35705950 and TERT rs2736100 variants among Turkish IPF patients. Additionally, we assessed associations between these genetic variants and clinical parameters including gender-age-physiology (GAP) score, percent predicted forced vital capacity (FVC%), percent predicted diffusing capacity for carbon monoxide (DLCO%), and the presence of honeycombing on high-resolution computed tomography (HRCT).

**Results:**

The allele frequency of the TERT rs2736100 variant showed no significant difference between IPF patients and healthy controls (41.7% vs. 43.7%, OR = 0.92, *p* = 0.73). Conversely, the allele frequency of the MUC5B rs35705950 variant was significantly higher in IPF patients compared to controls (39.6% vs. 12%, OR = 4.81, *p* = 0.0001). IPF patients carrying the homozygous MUC5B variant (TT) exhibited significantly higher mean FVC% values than those without the variant (GG) (82.2% vs. 71.7%, respectively; *p* = 0.004). Furthermore, the mean age at diagnosis was significantly older in IPF patients carrying at least one T allele of the MUC5B variant (GT + TT) compared to non-carriers (GG) (67.7 years vs. 62.3 years, respectively; *p* = 0.013).

**Conclusions:**

Our findings indicate that the MUC5B rs35705950 variant is significantly associated with increased IPF susceptibility among Turkish patients. In contrast, the TERT rs2736100 variant was not linked to IPF risk. Additionally, the presence of the MUC5B rs35705950 variant correlated with later disease onset and relatively preserved pulmonary function in this patient population.

**Supplementary Information:**

The online version contains supplementary material available at 10.1186/s12890-025-03903-7.

## Introduction

Idiopathic pulmonary fibrosis (IPF) is a progressive and fatal fibrotic lung disease with unknown etiology. Although two novel antifibrotic drugs currently available can slow disease progression and modestly extend survival, they do not provide a cure. Consequently, IPF typically advances to end-stage lung disease, eventually necessitating lung transplantation [1]. Given the limitations of antifibrotic therapies, which cannot reverse established fibrotic changes, early identification of individuals at genetic risk is crucial. Early detection may enable initiation of antifibrotic treatments at less advanced disease stages, potentially improving patient outcomes.

Recent genetic research has identified several genetic variants associated with increased susceptibility to IPF. Genome-wide association studies (GWAS) have highlighted genetic variations in the MUC5B gene as potential contributors to IPF development [2]. Among these, the rs35705950 variant located in the promoter region of the MUC5B gene has been extensively studied and is known to upregulate MUC5B gene expression [3]. Elevated levels of MUC5B protein have been observed in the distal airways of IPF patients [4]. This overexpression is hypothesized to contribute to lung injury and inflammation, processes implicated in IPF pathogenesis.

Previous studies examining patient survival indicate that the presence of the MUC5B rs35705950 variant is associated with improved survival outcomes in IPF patients [5]. Additionally, respiratory function impairment severity has been correlated with the rs35705950 variant in patients with interstitial lung disease (ILD) [6].

Recent findings further demonstrate that IPF patients treated with antifibrotics who carry the MUC5B rs35705950 T allele exhibit longer survival compared to non-carriers [7]. These observations collectively suggest that the MUC5B rs35705950 variant may influence clinical manifestations and disease progression in IPF.

The genetic etiology of IPF has also been linked to telomere length. In both sporadic and familial IPF cases, several studies have identified telomere shortening as a risk factor, and functional variants in telomere maintenance-related genes have been frequently reported [8]. Telomere dysfunction is thought to contribute to IPF pathogenesis through cellular aging and senescence in bronchoalveolar epithelial cells. A common variant (rs2736100) in the telomerase reverse transcriptase (TERT) gene was recently associated with IPF susceptibility [9]. Higher allele frequency of the rs2736100 variant, located in the second intron of the TERT gene, has been reported in IPF patients compared to control subjects [10]. Furthermore, both TERT rs2736100 and MUC5B rs35705950 variants have been associated with ILD [11, 12].

However, some studies present contradictory findings regarding these genetic variants. For instance, in subjects with interstitial lung abnormalities (ILA), the MUC5B rs35705950 variant was associated with both ILA and IPF, whereas the TERT rs2736100 variant showed no association [13]. Similarly, two other cohort studies did not find significant associations between the TERT rs2736100 variant and IPF [14, 15].

Additionally, some populations exhibited weaker associations between the MUC5B rs35705950 variant and IPF than previously reported, possibly due to population-specific genetic backgrounds or environmental exposures [16, 17]. In a Chinese cohort, neither of these variants showed associations with IPF [18]. Moreover, a recent study investigating the impact of the MUC5B variant on IPF survival outcomes reported no significant association [19]. Together, these data suggest that the distribution and clinical relevance of MUC5B and TERT variants in IPF may differ across populations.

Given these inconsistencies, it remains unclear whether MUC5B and TERT variants are associated with IPF susceptibility among Turkish patients. To our knowledge, there have been no previous studies examining the distribution and clinical implications of these variants in Turkish IPF patients. Thus, this study aims to investigate the frequency of MUC5B and TERT variants in Turkish IPF patients and analyze their associations with clinical characteristics, including potential contributions to early risk stratification and prognosis.

## Material and method

### Participants

This study was conducted between November 2018 and October 2019 at Akdeniz University Faculty of Medicine, Department of Chest Diseases. Patients diagnosed with IPF based on the 2018 American Thoracic Society (ATS)/European Respiratory Society (ERS) Clinical Practice Guidelines and healthy control volunteers were included in the study [20]. The healthy control group consisted of individuals matched with the IPF cohort for age and sex, who had no respiratory symptoms or abnormal physical examination findings suggestive of ILD, such as clubbing or velcro crackles, and had normal chest radiographs. Pulmonary function tests were not performed in this group. Written informed consent was obtained from all participants. The Clinical Research Ethics Committee of Akdeniz University Faculty of Medicine approved the study on 21 November 2018 (Decision Number: 816). The study was conducted in full accordance with the ethical principles outlined in the Declaration of Helsinki. Financial support for the study was provided by the Akdeniz University Scientific Research Projects Coordination Unit (Project Number: TTU-2019-4793).

## Clinical features

Demographic data (age, gender, smoking history) were collected from both IPF patients and control subjects. Pulmonary function tests and helium dilution tests were performed on IPF patients, and blood samples for genetic analyses were simultaneously obtained from both groups. The distribution of MUC5B and TERT gene variants was examined in each group. Associations between these genetic variants and clinical features at the time of diagnosis—including gender-age-physiology (GAP) score, percent predicted forced vital capacity (FVC%), percent predicted diffusing capacity for carbon monoxide (DLCO%), and high-resolution computed tomography (HRCT) findings (presence or absence of honeycombing)—were evaluated.

## Genotyping

Genomic DNA was extracted from blood samples using the silica column method (Exgene Blood SV Mini, GeneAll, Korea). Genotyping of the TERT (NM_198253.2: c.1574-3777G > T, NC_000005.10: g.1286401 C > A, rs2736100) and MUC5B (NM_002458.2: c.−3133G > T, NC_000011.10: g.1219991G > T, rs35705950) variants was performed using quantitative PCR with allele-specific primers (LightSNiP Assay, TIB MOLBIOL, Germany). Allelic discrimination was conducted through melting curve analysis using fluorescent probes specific to the alleles. Genotype analyses were conducted using a LightCycler 480 II instrument (Roche Molecular Systems, Inc.) and analyzed with LightCycler 480 SW1.5 software at Akdeniz University Health Sciences Research Application Center (SBAUM). For the evaluation of the MUC5B gene g.1219991G > T (rs35705950) variant, the “T” allele was designated as the risk allele. Genotypes were categorized as GG (wild-type), GT (heterozygous), and TT (homozygous). Similarly, for the TERT gene g.1286401 C > A (rs2736100) variant, the “A” allele was defined as the risk allele, with genotypes classified as CC (wild-type), CA (heterozygous), and AA (homozygous).

### Statistical analysis

Statistical analyses were performed using IBM SPSS Statistics 23 and GraphPad Prism software. Descriptive statistics included frequencies (number, percentage) for categorical variables and means with standard deviations for numerical variables. Comparisons of continuous variables between two groups were made using the Mann-Whitney U test and among more than two groups using the Kruskal-Wallis test. Chi-square or Fisher’s exact tests were employed to assess associations between categorical variables. A p-value of less than 0.05 was considered statistically significant.

## Results

### General characteristics of patients and control subjects

Genetic and clinical characteristics were assessed in 96 patients with IPF (mean age 67.7 ± 8.5 years) and 71 age-matched healthy control subjects (Table 1).

**Table 1:** Demographic features, respiratory functions and radiological findings of participants

Characteristic	Control	IPF
	*n* = 71	*n* = 96
**Age**,** mean ± SD**	67.6 ± 6.4	67.7 ± 8.5
**Age at diagnosis**,** mean ± SD**	-	65.9 ± 8.7
**Gender (Female)**	19%	32%
**Smoking history**	49%	72%*
**Smoking amount (P/Y)**,** mean ± SD**	36.6 ± 14.1	32 ± 17.5
**FVC (%predicted)**,** mean ± SD**	-	72.1 ± 16.3
**DLCO (%predicted)** (*n* = 89),** mean ± SD**	-	59.8 ± 15.9
**GAP score**,** median (min-max)**	-	3 (0–8)
**HRCT (with honeycombing)**	-	%72

IPF = Idiopathic Pulmonary Fibrosis; P/Y = Packet/Year; SD = Standard Deviation; FVC = Forced Vital Capacity; % pred. = Percent of predicted value; DLCO = Diffusing capacity of the lung for carbon monoxide; GAP = Gender-Age-Physiology index; min = minimum; max = maximum; HRCT = High-Resolution Computed Tomography

*p = 0.004,* smoking rate in IPF versus Control group (Chi-Square test)*

In both groups, the majority of participants were male. The proportion of smokers was significantly higher in the IPF group compared to the control group (72% vs. 49%, *p* = 0.04), although the mean number of cigarettes smoked was similar between groups. The mean age at IPF diagnosis was 65.9 ± 8.7 years. The mean percent predicted FVC and DLCO values were 72.1 ± 16.3 and 59.8 ± 15.9, respectively. The median GAP score was 3 (range: 0–8). Honeycombing on HRCT was observed in 72% of IPF patients.

In contrast, the MUC5B rs35705950 variant demonstrated a markedly higher allele frequency in IPF patients compared to controls (39.6% vs. 12%), corresponding to a significantly increased disease risk (OR = 4.81, 95% CI: 2.68–8.63, p < 0.0001).

**Fig. 1:**
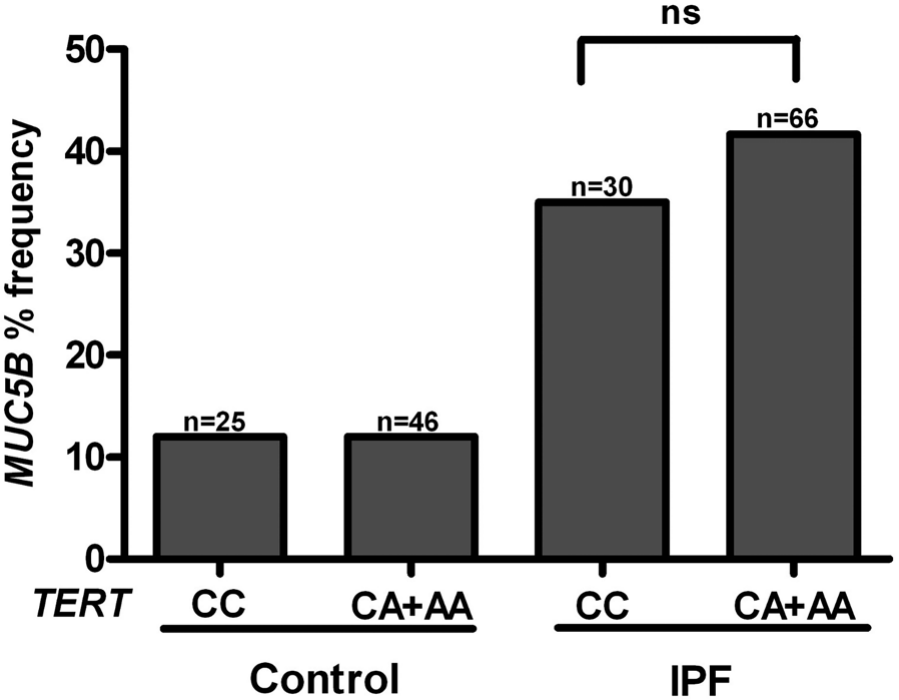
Distribution of the MUC5B rs35705950 variant frequency according to TERT rs2736100 risk allele status. The frequency of the MUC5B variant was comparable between carriers (CA + AA) and non-carriers (CC) of the TERT risk allele within both IPF and control groups. However, the frequency of the MUC5B variant was significantly higher in IPF patients compared to controls, regardless of TERT variant status (*p* = 0.0001 and *p* = 0.007, respectively) However, the MUC5B rs35705950 variant frequency remained significantly higher in the IPF group compared to the control group, regardless of TERT rs2736100 variant status (41.7% vs. 12%, *p* = 0.0001 and 35% vs. 12%, *p* = 0.007, respectively). When the TERT rs2736100 variant distribution was assessed in relation to the presence or absence of the MUC5B rs35705950 variant, no significant differences were observed in either the IPF or control groups (Fig. 2)

 To evaluate the interaction between these two variants, the distribution of MUC5B rs35705950 alleles was compared between individuals with and without the TERT rs2736100 variant. In both the IPF and control groups, the frequency of the MUC5B variant was similar among individuals carrying the TERT risk allele (CA+AA) and those without it (CC). However, within both TERT-defined subgroups, the MUC5B variant was significantly more frequent in IPF patients compared to controls (p = 0.0001 and p = 0.007, respectively; Figure 1).

However, in patients with the TT genotype, the mean FVC was significantly higher than in those with the GG genotype (82.2% vs. 71.7%, p = 0.004). Additionally, the mean age at diagnosis was significantly higher in patients with GT and TT genotypes compared to those with the GG genotype (67.7 vs. 62.3 years, p = 0.013).

To further elucidate whether genetic variant frequencies differed according to the age at diagnosis, IPF patients were stratified into two subgroups based on age: ≤60 years (early-onset) and >60 years (late-onset), in accordance with previous literature examining age-related clinical and genetic heterogeneity. Age at diagnosis, a standardized and objectively ascertainable time point, was utilized for classification in all participants. While the frequency of the TERT rs2736100 variant did not differ significantly between these age-based subgroups, the MUC5B rs35705950 variant was significantly more prevalent in the late-onset group compared to the early-onset group (45.7% vs. 24.1%, p = 0.008) (Table 2).

**Table 2:** Characteristics of IPF patients according to the age at diagnosis

	Early ≤ 60 years (*n* = 27)	Late > 60 years (*n* = 69)	*P* value*
**Gender (Female)**	11%	17%	ns
**Smoking history**	81%	68%	ns
**FVC** (%predicted) (mean ± SD)	68.13 ± 16.69	73.75 ± 16.03	ns
**DLCO** (%predicted) (mean ± SD)	58.74 ± 17.06	60.39 ± 15.50	ns
**GAP score > 3 (%)**	22%	54%	0.005
***TERT*** **(% risk allele)**	42.6	41.3	ns
***MUC5B*** **(% risk allele)**	24.1^a^	45.7^b^	0.008
**HRCT with honeycombing (%)**	52%	80%	0.011

IPF = Idiopathic Pulmonary Fibrosis; n = number; ns = not significant. p-values were calculated using Fisher’s Exact test. ^a^*p* = 0.046 vs. Control; ^b^*p* < 0.001 vs. Control

IPF patients diagnosed at ≤60 years of age showed a significantly higher frequency of the MUC5B rs35705950 variant compared to the control group. No significant differences were observed between early- and late-onset IPF patients in terms of gender, smoking history, FVC, or DLCO. However, patients diagnosed after the age of 60 exhibited higher GAP scores and a greater frequency of honeycombing on HRCT compared to those diagnosed earlier.

### Allele frequency distributions

The allele frequency of the TERT rs2736100 variant was similar between IPF patients and healthy controls (41.7% vs. 43.7%, OR = 0.92, 95% CI: 0.59–1.42, *p* = 0.73), indicating no significant association with disease status (Table 3).

**Table 3:** Distribution of TERT and MUC5B variants among IPF patients and control subjects

	Control (*n* = 71)		IPF (*n* = 96)	
TERT Genotype	Number	Percent	Number	Percent
CC	25	35%	30	31%
CA	30	42%	52	54%
AA	16	23%	14	15%
**AF % risk allele**	**43.7**		**41.7**	
*p- value* OR (95% CI)	0.73 0.92 (0.59–1.42)			

MUC5B Genotype	Number	Percent	Number	Percent
GG	56	79%	32	33%
GT	13	18%	52	54%
TT	2	3%	12	13%
**AF % risk allele**	**12**		**39.6**	
*p- value* OR (95% CI)	< 0.0001 4.81 (2.68–8.63)			

IPF = Idiopathic Pulmonary Fibrosis; n = number; AF = Allele Frequency; OR = Odds Ratio; CI = Confidence Interval. p-values were calculated using the Chi-square test

**Fig. 2:**
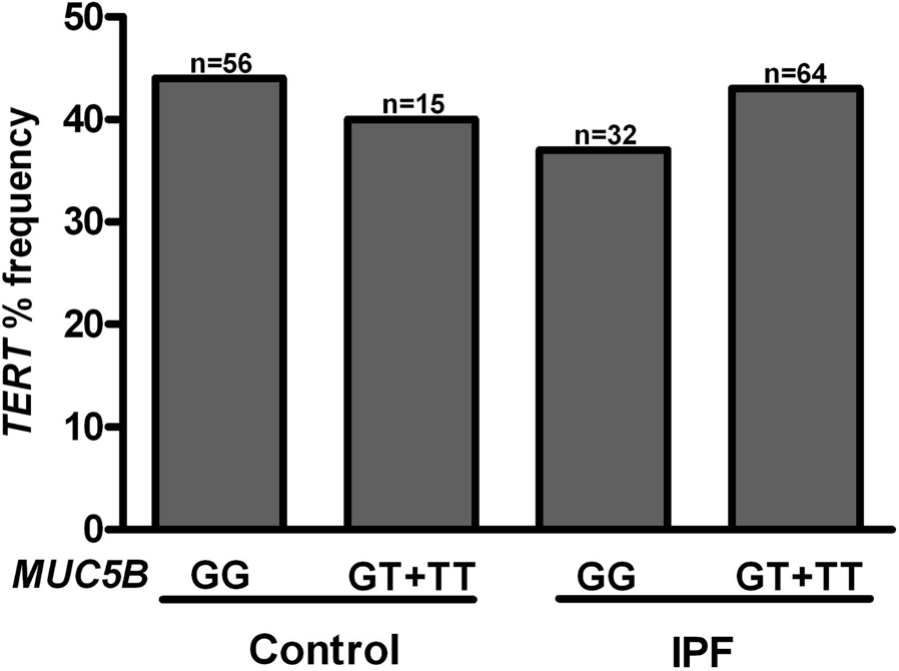
Distribution of TERT rs2736100 variant frequencies based on the presence or absence of the MUC5B rs35705950 risk allele (GT + TT) in IPF and control groups. No significant differences were observed between the IPF and control groups

Since the proportion of smokers was higher in the IPF group, we further investigated whether the distribution of the MUC5B rs35705950 variant differed according to smoking status. The analysis revealed no significant difference in MUC5B variant frequency between smokers and non-smokers in either the IPF or control groups (Supplementary Table 1).

### Clinical Variables

Given the increased frequency of the MUC5B rs35705950 variant in the IPF group, we evaluated whether this variant was associated with specific clinical features of the disease. No significant differences were found among the MUC5B genotype groups (GG, GT, TT) in terms of FVC, DLCO, GAP score, or presence of honeycombing on HRCT (Table 4).

**Table 4:** Clinical features of IPF patients relative to *MUC5B* rs35705950 genotypes

	GG (*n* = 32)	GT (*n* = 52)	TT (*n* = 12)	*p*-value*
**Age at diagnosis**,** (mean ± SD)**	62.3 ± 8.8	67.7 ± 8.3	67.7 ± 7.5	0.013**
**FVC (% predicted) (mean ± SD)**	71.7 ± 18.3	70.1 ± 15.8	82.2 ± 8.4	ns
**DLCO (% predicted) (mean ± SD)**	58.6 ± 17.4	61.7 ± 13.8	56.3 ± 19.8	ns
**GAP score (median**,** min-max)**	3 (0–5)	3 (1–8)	3 (1–5)	ns
**HRCT honeycombing (** %)	69%	71%	83%	ns

n = number; ns = not significant. *p-values were calculated using the Kruskal-Wallis test. **p = 0.013 for GG vs. GT + TT (Mann-Whitney U test); ⁑ p = 0.004 for GG vs. TT (Mann-Whitney U test)

## Discussion

Idiopathic pulmonary fibrosis (IPF) is a progressive fibrotic lung disease, and early diagnosis and timely initiation of treatment are crucial for improving clinical outcomes. A deeper understanding of genetic risk factors associated with IPF may facilitate the identification of at-risk individuals. Early genome-wide association studies identified common variants in the MUC5B and TERT genes as potential contributors to IPF susceptibility. However, subsequent investigations reported inconsistent findings, suggesting that these associations may vary across different populations.

 In this study, we assessed the frequency of the MUC5B rs35705950 and TERT rs2736100 variants in a Turkish cohort of patients with IPF and explored their association with clinical characteristics. Our findings demonstrated that the MUC5B rs35705950 variant was significantly more prevalent in the IPF group compared to healthy controls. In contrast, the TERT rs2736100 variant showed no significant association with IPF in our cohort. The presence of the MUC5B rs35705950 variant was associated with an over four-fold increased risk of IPF.

 Additionally, our data showed that IPF patients carrying the MUC5B rs35705950 variant were diagnosed at an older age and exhibited better -preserved lung function compared to non-carriers. This suggests a potential modifying effect of the MUC5B variant on disease phenotype. Furthermore, analysis of the combined influence of both variants revealed that the inclusion of the TERT rs2736100 variant did not enhance the predictive association observed with MUC5B rs35705950 alone. These results underscore the importance of population-specific studies and highlight the clinical relevance of the MUC5B rs35705950 variant in the Turkish IPF population.

 Previous studies investigating the relationship between the MUC5B rs35705950 (T/G) variant and IPF risk have yielded divergent results across populations. An initial GWAS identified the minor T allele, located approximately 3 kb upstream of the MUC5B transcription start site on chromosome 11p15, as significantly associated with IPF susceptibility [2]. The study reported that heterozygous carriers (GT) had a 9-fold increased risk, while homozygous carriers (TT) had a 21.8-fold increased risk of developing IPF. Multiple subsequent independent cohorts have replicated these findings, consistently demonstrating a strong association between MUC5B rs35705950 and IPF [5, 10, 21–23].

 However, the strength and presence of this association have varied across ethnic groups. While the association was observed in the German population, it was weaker in the Japanese cohort and absent in Korean and Chinese populations [16, 17]. A recent meta-analysis of studies conducted in various racial groups emphasized that this variant’s contribution to IPF risk may be population-specific. It concluded that Caucasians carrying the minor T allele were more susceptible to IPF than Asians, and that the TT genotype conferred a higher risk than the GT genotype [24].

 Our results are consistent with findings reported from European and North American Caucasian populations. In our Turkish cohort, the MUC5B rs35705950 variant was significantly associated with IPF, conferring more than a four-fold increase in disease risk. These findings reinforce the relevance of this variant in the pathogenesis of IPF within the Turkish population and further support the need for ethnicity-focused genetic research in fibrotic lung diseases. Telomere maintenance genes have been implicated in the fibrotic processes of lung tissue, and several studies have explored their association with IPF development [25]. Despite this, our study did not identify a significant difference in the frequency of the TERT rs2736100 A allele between IPF patients and control subjects. While some earlier reports have demonstrated a link between the rs2736100 variant and IPF, others—consistent with our findings—have reported no such association [9–11, 14].

Furthermore, analyses evaluating the combined effects of MUC5B and TERT variants on IPF risk have indicated that, although MUC5B rs35705950 is a strong individual risk factor for IPF, the inclusion of TERT rs2736100 does not confer additional predictive value [15, 26]. This aligns with our results, which showed no enhancement of the association when both variants were considered together. These findings further emphasize the dominant role of the MUC5B rs35705950 variant in IPF susceptibility in the Turkish population and support prior evidence questioning the independent significance of TERT rs2736100 in certain populations.

 The presence of the MUC5B rs35705950 variant has also been associated with better preserved pulmonary function and prolonged survival in IPF patients [5]. Although some studies have not confirmed an association with pulmonary function measures such as FVC and DLCO, others have reported that carriers of the T allele exhibit higher FVC values [5, 16, 22]. A recent study further supported this finding, showing a strong correlation between the T allele and FVC levels [7]. In line with these findings, our results demonstrated that patients with the TT genotype had significantly higher FVC values, while DLCO values did not differ across genotypes. This suggests a modest positive effect of the rs35705950 T allele on FVC.

 In addition to pulmonary function, the MUC5B rs35705950 variant has been linked to delayed disease onset and improved survival. Several studies have reported that carriers of the variant tend to be older at diagnosis and experience slower disease progression compared to non-carriers [5, 15, 27, 28]. Our findings are consistent with this, as IPF patients with the MUC5B variant were significantly older at diagnosis, with the highest frequency observed in patients diagnosed after the age of 60 years.

 Emerging evidence from studies on relatives of IPF patients also supports a potential age-dependent association. One study reported that 14% of at-risk relatives had early interstitial abnormalities on HRCT at a mean age of 50 years, with the frequency of the MUC5B variant approximately two-fold higher than in controls, though still lower than in IPF patients [29]. Another recent study of relatives from familial interstitial pneumonia (FIP) cohorts found that those with preclinical pulmonary fibrosis (mean age ~66 years) had a higher frequency of the MUC5B rs35705950 variant than relatives without abnormalities on HRCT [26].

 Taken together, these findings suggest an age-related association between the MUC5B rs35705950 variant and the prevalence of pulmonary fibrosis. Further studies are warranted to clarify the temporal dynamics of this variant’s influence on IPF risk, particularly in the context of age at diagnosis. The presence of honeycombing in a subset of patients is consistent with the fact that IPF is often diagnosed at an advanced stage due to its insidious onset and progressive nature. According to current guidelines, honeycombing is considered a radiologic marker of irreversible fibrosis and is a characteristic feature of the usual interstitial pneumonia (UIP) pattern, indicative of advanced-stage disease. Previous studies have also linked the MUC5B rs35705950 variant to radiological findings, such as honeycombing on HRCT. Specifically, IPF patients carrying the variant were reported to be more likely to exhibit honeycombing patterns [12, 30]. Similarly, in patients with chronic hypersensitivity pneumonitis (cHP), the rs868903 variant of MUC5B was associated with fibrosis extent [31]. Additionally, in relatives of familial interstitial pneumonia (FIP) patients with fibrotic abnormalities, the MUC5B variant was more common among those with higher fibrosis scores than in those with lower scores [26].

 In our cohort, although the severity of fibrosis was not quantitatively assessed, we did not detect an association between the presence of honeycombing and the MUC5B rs35705950 variant. These findings suggest that the relationship between this variant and the extent or severity of fibrosis is complex and may likely be influenced by additional genetic or environmental factors. Given that we did not perform detailed radiological classification of HRCT findings according to the UIP classification system (e.g., definite UIP, probable UIP, indeterminate for UIP), we cannot comment on potential associations between genetic variants and specific UIP sub-patterns. While the MUC5B rs35705950 variant increases IPF risk and is associated with older age at disease onset and a more favorable prognosis, our findings underscore the need for further studies investigating interactions between this variant and other pathogenic genetic variants.

### Limitations

This study has several limitations. First, pulmonary function tests were not performed in the control group; although control subjects were carefully selected, this may have led to the overlooking of subclinical or asymptomatic interstitial lung disease. Second, detailed radiological classification of HRCT findings according to the UIP classification system was not conducted, limiting our ability to assess associations between genetic variants, specific UIP sub-patterns, and the degree of fibrosis. Third, due to the cross-sectional design, retrospective data on initial symptom onset and the first detection of HRCT abnormalities were not available, and therefore patients' age at diagnosis was used as a proxy. This constrains the precision of age-based subgroup analyses. Finally, larger, multicenter cohort studies incorporating comprehensive genomic analyses are required to validate and expand upon our findings.

## Conclusion

In conclusion, our findings indicate that the MUC5B rs35705950 variant is significantly associated with increased IPF susceptibility in the Turkish population, whereas the TERT rs2736100 variant does not appear to confer IPF risk. The MUC5B variant was associated with older age at diagnosis and relatively better-preserved lung function, highlighting its potential role as a modifier of IPF clinical phenotype. Further research is needed to elucidate the precise mechanisms through which MUC5B influences disease onset and progression.

## Supplementary Information

Supplementary Material 1

## Data Availability

The datasets generated and/or analysed during the current study are available in the figshare repository, [https://doi.org/10.6084/m9.figshare.29205776.](https:/doi.org/10.6084/m9.figshare.29205776.)
