# Supplementary material for: The distribution and the clinical importance of MUC5B and TERT variants in Turkish patients with idiopathic pulmonary fibrosis
Source: BMC Pulm Med. 2025 Sep 25;25:429. doi: 10.1186/s12890-025-03903-7 (PMC12465160; doi:10.1186/s12890-025-03903-7)
Supplement: Supplementary file 1 — Supplementary Material 1 [file 12890_2025_3903_MOESM1_ESM.docx]

**Supplementary Table 1.** TERT and MUC5B variant frequency by smoking status.

|  | **Control** | | | **IPF** | | |
| --- | --- | --- | --- | --- | --- | --- |
|  | **Smokers**  **(n=35)** | **Not Smokers**  **(n=36)** | ***P-value*** | **Smokers**  **(n=69)** | **Not Smokers**  **(n=27)** | ***P-value*** |
| ***TERT* (% risk allele)** | 40.3 | 47.1 | ns | 42.6 | 41.3 | ns |
| ***MUC5B*  (% risk allele)** | 9.7 | 14.3 | ns | 42.6 | 38.4 | ns |

Abbreviations: IPF = Idiopathic Pulmonary Fibrosis; n = Number; ns = Not significant. p-values were calculated using Fisher’s Exact test.
